# Trusted strangers: social affordances for social cohesion

**DOI:** 10.1007/s11097-017-9554-7

**Published:** 2017-12-29

**Authors:** Erik Rietveld, Ronald Rietveld, Janno Martens

**Affiliations:** 1 AMC/Department of Philosophy/ILLC/Amsterdam Brain & Cognition, University of Amsterdam, Amsterdam, The Netherlands; 2 RAAAF, Westerdok 744, 1013BV Amsterdam, The Netherlands; 3 AMC, University of Amsterdam, Amsterdam, The Netherlands

**Keywords:** Enactive architecture, Visual art, Social affordances, Radical embodied cognitive science, Material engagement theory, Skilled intentionality, Public domain, Metaplasticity

## Abstract

How could the paradigm shift towards enactive embodied cognitive science have implications for society and politics? Translating insights form enactive embodied cognitive science into ways of dealing with real-life issues is an important challenge. This paper focuses of the urgent societal issue of social cohesion, which is crucial in our increasingly segregated and polarized Western societies. We use Rietveld’s (2016) philosophical Skilled Intentionality Framework and work by the multidisciplinary studio RAAAF to extend Lambros Malafouris’ Material Engagement Theory (2013) to the social domain. How could a landscape of *social affordances* generate change in the behavioral patterns of people from different socio-cultural backgrounds? RAAAF is currently imagining and planning an ambitious intervention in the public domain that could really change existing socio-cultural practices and aims to contribute to social cohesion. An animation film it made introduces a landscape of social affordances. We will present and discuss this *Trusted Strangers* animation film, which is a thinking model for new public domain all over the world. Tha animation film visualizes how a well-designed landscape of social affordances could invite all sorts of interactions between people from different socio-cultural backgrounds.

## Introduction

Up until now, the philosophy of embodied and enactive cognition – judged on the basis of the published body of work – has been primarily an important theoretical exercise. This raises the question of how the paradigm shift towards enactive embodied cognitive science could not just change ideas in academia but also have impact for society and politics. Think of challenges such as the transition to using more sustainable sources of energy; increasing social cohesion (crucial in our increasingly segregated and polarized Western societies); realizing universal access to food, water and housing; increasing the inclusivity of society; preserving cultural heritage; and developing healthier lifestyles. Translating insights form enactive embodied cognitive science into ways of dealing with these real-life issues is an important challenge.

How could one for example extend Lambros Malafouris’ Material Engagement Theory (MET) (2013) to the social domain of everyday life? We believe that this would require both theoretical work on intentionality and creative work to imagine societies that can improve on the weaknesses of our current ones. With respect to theory, Malafouris’ (2013, 2014) discussion of material engagement clearly opens up this possibility. To use his own words:

“[T]he priority of material engagement seems quite natural in view of what we know in archaeology and anthropology about the profound way *materiality* envelops our everyday lives and *mediates our social ways of being with one another*.” (Malafouris 2014, pp. 140-141, our italics)

Malafouris’ insight that the material mediates social ways of being is crucial, because it implies that by changing the material, we can change our socio-cultural practices. On the basis of their theoretical extension of Material Engagement Theory to the social domain, Gallagher and Ransom (2016) argue that “material artefacts afford the possibility for coordinating social forces in ways that might not otherwise be possible, and in this regard there are cases where specific kinds of material engagement can disclose new *social affordances*.” (Gallagher and Ransom 2016, p. 349, our italics).

Affordances are the possibilities for action provided by the environment (Gibson 1979; Chemero 2003; Rietveld and Kiverstein 2014). Social affordances are a subcategory of affordances: possibilities for social interaction or sociability provided by the environment.^1^ Just like our brains, socio-cultural practices are *plastic*: they change as a result of what we do and make; as a result of the way we engage with affordances (Malafouris 2013, 2014; Rietveld et al. 2014, 2015). Social affordances can invite social interactions that over time, if engaged with by sufficient amounts of people, may result into transformed patterns of behavior; i.e. into transformed socio-cultural practices (Van Dijk and Rietveld 2017). This kind of plasticity is a unique trait of the human form of life. To use Malafouris’ words: “The mind's extraordinary plasticity and its reciprocal openness to cultural influence and variation through active engagement with the material world are […] the keys to understanding the distinctive features of human cognition and how it changes” (Malafouris 2013, 46). His notion of metaplasticity (Malafouris 2009, 2010, 2013, 2015; Malafouris and Renfrew 2008) – which within the context of MET characterizes the “emergent properties of the enactive constitutive intertwining between brain and culture” (Malafouris 2013, 46) – is helpful in developing a perspective on generating the behavioral change that is needed for dealing with societal challenges like the ones listed above.

MET shows convincingly that material aspects of the environment can influence the development of activities on multiple time scales, from the very short millisecond time scales of neural activity, to the longer timescales of situated activities by individuals, up to the very long timescales of large-scale processes like development and (cultural) evolution (Malafouris 2015; Rietveld 2008b, c). The persistent character of the material world (think for example of environmental aspects like buildings,^2^ lakes and rivers) offers a “temporal anchoring […] that helps us to move and think across the scales of time. When humans engage the material world they establish a bridge with the larger-scale processes at work […] beyond their control which are embodied in the objects at hand. With things past becomes present.” (Malafouris 2015, p.15).

One specific case in which material engagement discloses new social affordances is in the work of architects. Architects are experts in creating new affordances through alterations of our material environment. The Amsterdam-based multidisciplinary studio RAAAF [Rietveld Architecture-Art-Affordances] has developed an affordance-based design approach. Some of its artworks have the ambition to change entire socio-cultural practices (Rietveld and Brouwers 2016; Rietveld 2016; Rietveld et al 2014, 2015). As indicated by its full name above, RAAAF starts from philosophical work on affordances and skilled intentionality (Rietveld 2008a, b; Rietveld and Kiverstein 2014; Rietveld 2016; Rietveld and Brouwers 2016; Rietveld, Denys and Van Westen, in press).

Skilled intentionality is defined as coordination with multiple affordances simultaneously (Rietveld, Denys and Van Westen, in press). The Skilled Intentionality Framework understands forms of skilled action (broadly understood), including skilled *social* interaction, primarily as situated in and engaging with a rich landscape of affordances that includes possibilities for social interaction (Rietveld and Kiverstein 2014; Rietveld and Brouwers 2016, footnote 1; cf. Gallagher and Ransom 2016).

The Skilled Intentionality Framework takes the dynamics of the *entire system* ‘brain-body-landscape of affordances’ as its starting point. It is here that skilled intentionality converges with Malafouris’ analysis of material engagement. Take for example his analysis of creativity in the context of craftsmanship in pottery:

“To understand these complex dynamics, we need to rethink what happens in the brain when people are acting creatively in terms of the radical embodied cognitive science that aims to *integrate the whole system ‘brain–body–landscape of affordances’* (Bruineberg and Rietveld 2014; Rietveld and Kiverstein 2014)” (Malafouris 2014, p. 147, our italics).

As far as intentionality is concerned, MET is thus like SIF in emphasizing engagement with the rich landscape of affordances offered by the material and social environment. The same questions about how to make the framework tangible and concrete enough to deal with societal issues can be raised with respect to both frameworks. Metaplasticity is a valuable addition to SIF because it helps us to think about changing practices over longer periods of time, which is relevant for dealing with societal issues. But how could changing the practices be realized in a concrete way by changing the available affordances? In what follows we will primarily use the affordance-based SIF vocabulary explore this.

**Fig. 1:**
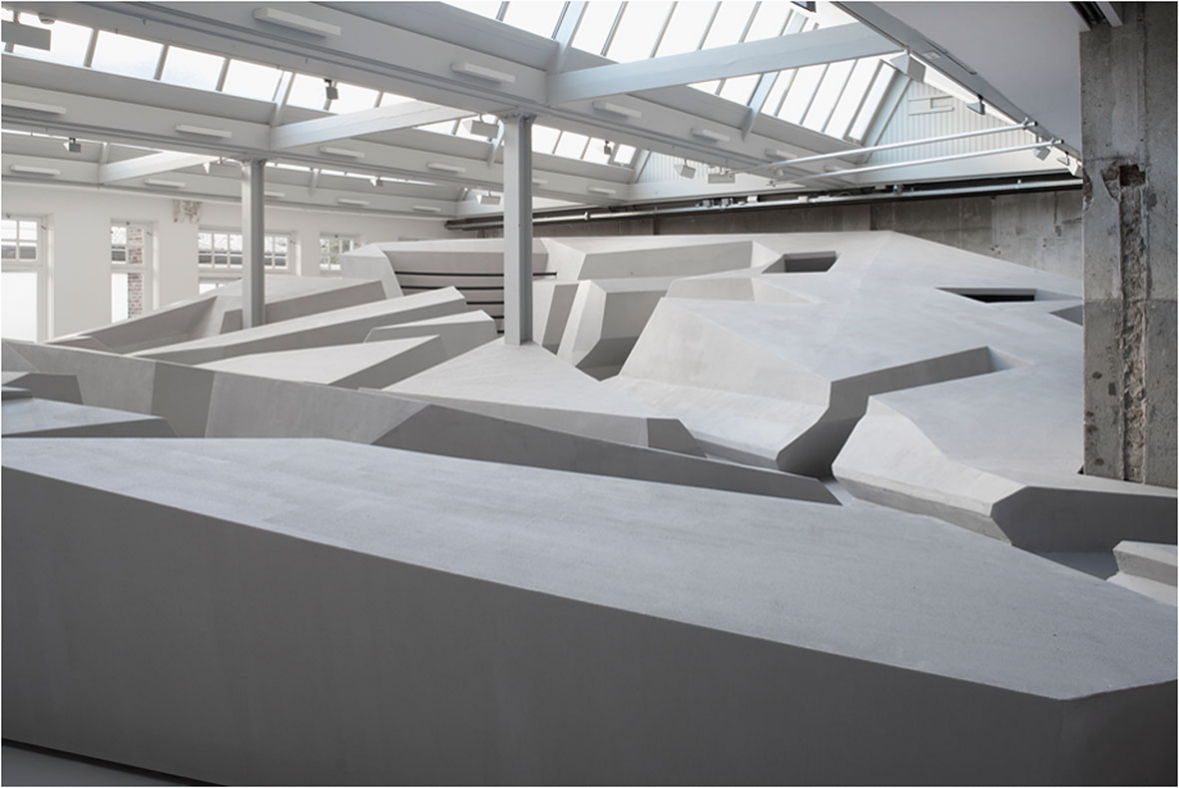
*The end of sitting* by RAAAF | Barbara Visser. Photo: Jan Kempenaers

**Fig. 2:**
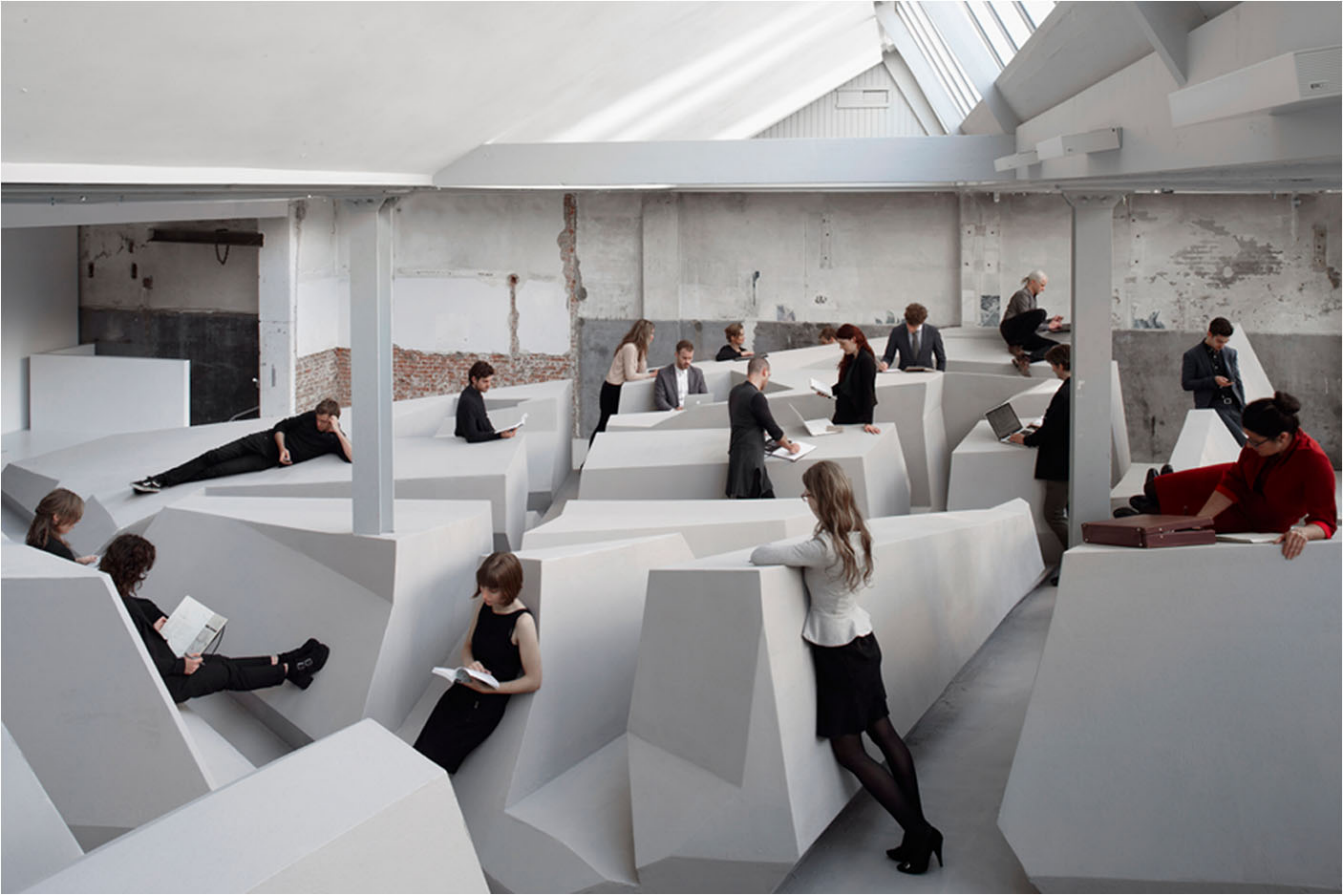
*The end of sitting* by RAAAF | Barbara Visser. Photo: Jan Kempenaers

To make this skilled intentionality-based approach material and tangible, RAAAF and visual artist Barbara Visser have built the large architectural art installation The End of Sitting in an art gallery in the center of Amsterdam: Looiersgracht 60. The End of Sitting (Figs. 1 and 2) is both a landscape of affordances and a life-sized physical thinking model for imagining the possibility of a more active way of living in 2025 (Rietveld 2016; Withagen and Caljouw 2015). Empirical research by Withagen and Caljouw (2015) showed that The End of Sitting might be able to generate behavioural change in real-life situations by offering an innovative local landscape of affordances that supports standing – though more studies are needed to investigate its impact, in particular over longer periods of time and with older subjects.

How could a landscape of *social* affordances generate change in the behavioral patterns of people from different socio-cultural backgrounds? RAAAF is currently imagining and planning another ambitious intervention, this time in the public domain, that could also change existing socio-cultural practices and aims to contribute to social cohesion. In the remainder of this paper we will discuss the societal urgency of taking care of the public domain (Section 2), and the roles familiarity and trust play in such efforts (Section 3). In Section 4 we present and discuss the Trusted Strangers animation film, which is a thinking model for new public domain. This film aims to visualize how a well-designed landscape of (social) affordances could contribute to social cohesion. The premiere of the Trusted Strangers film took place at the Stedelijk Museum for Modern and Contemporary Art in Amsterdam in November 2017.

## The necessity of good public domain in the city of the twenty-first century

The Brexit referendum in the UK brought to light many divisions: between the older and younger generations, between Scotland, Northern Ireland and England, between city and countryside, and between higher and less educated parts of society. Meanwhile, across the Atlantic, Trump supporters seem to be living in a universe that is different from the one many liberal East- and West Coast dwellers and our US Facebook friends seem to be living in. Additionally, it is widely observed that many people are living “in their phones” and only get information that’s able to penetrate their specific social media “filter bubble”. All of these phenomena can be understood as reflecting segregation and a lack of social cohesion. Segregation, be it spatial or digital, tends to reduce the chances of hearing voices from people outside one’s own socio-cultural groups. Given that as a result of migration the Western societies will only increase in diversity the coming years, social cohesion – understood as the co-existence of disparities, *not* the elimination of particular backgrounds^3^ — will become one of the most important themes, which in turn means that the physical public domain will become more relevant than ever.

A public domain is a place where people from different socio-cultural groups can meet and sometimes actually do meet (Hajer and Reijndorp 2002). This important topic forces architects and urban designers to consider the fact that they shape more than just the physical environment — to a certain extent they also bear responsibility for the role that their designs have in shaping social reality. This fact is also stressed within MET, given its foundational theoretical position that “[i]n the human engagement with the material world, there are no fixed roles and clean ontological separations between agent entities and patient entities; rather, there is a constitutive intertwining between intentionality and affordance.” (Malafouris 2013, 149) Both in MET and in the SIF, intentionality is not to be located in the agent but is an aspect of the *entire system* ‘brain-body-landscape of affordances’ (Bruineberg and Rietveld 2014; Rietveld, Denys and Van Westen, in press). In other words, intentionality is an emergent property of this entire system. We cannot understand intentionality (and the collective patterns of behavior that form socio-cultural practices) without taking into account the affordances offered by the material environment. The observation that aspects of the material environment — like architectural designs — shape socio-cultural practices was in a sense also the starting point for the fascinating book *In Search of New Public Domain,* published in 2001 by Maarten Hajer and Arnold Reijndorp. Responding mostly to the urban challenges of the final decade of the previous century, this book now proves to still be as relevant as ever: creating new public domain will be one of the biggest challenges for city-making in the twenty-first century.

A good public domain is an elusive and complex phenomenon. The relevant qualities, problems and developments vary per neighborhood, city or region. Experience has taught RAAAF that it’s difficult to define universal conditions for the design of a good public domain. However, taking local differences seriously does not take away the importance of striving for better insight in deliberate interventions that could positively affect the social fabric of the city. As Hajer & Reijndorp put it:

“Designing public domain can then become a question of the stimulation of informal manifestations of diversity and the avoidance of interventions that are intended to make such manifestations impossible” (Hajer and Reijndorp 2002, 37)

So, a good public domain can generate or invite variety in the ways people do things and spontaneously express themselves; i.e. the spontaneous expression of diversity. If architects take this approach to public domain seriously, they will have to pay attention to the ways different people from diverse sociocultural backgrounds would engage and interact with a particular intervention, and incorporate this into their designs.

### RAAAF’s ambition of contributing to a well-functioning public domain

RAAAF [Rietveld Architecture-Art-Affordances], a multidisciplinary studio operating at the intersection of architecture, visual art and philosophy, was founded in 2006. Urgent societal issues often play an important role in the projects of our studio. Examples of recurring themes are increasing urbanization; the changing meaning of public space; issues related to extreme rainfall, droughts, or rising water levels of seas and rivers in The Netherlands; sustainability, the climate and ecology; and the potential of vacant cultural heritage. RAAAF’s strategic interventions can be radical and are often rooted in the historical background of a site or region, but add a new and legible interpretative layer to it. We developed the approach of ‘strategic interventions’: carefully chosen and precisely designed interventions that set a desired change in motion (Rietveld et al 2014). This can be at the level of an entire city or country, but its application might just as well be limited to a park or a site-specific art installation.

RAAAF wants to contribute to the investigation and creation of interesting and well-functioning public domain. We regard the study by Hajer and Reijndorp (2002) as providing a useful theoretic framework for further artistic research. We therefore actively investigate the possibilities of interventions that are attractive to different user groups, varying not just in ethnic background, but also in subcultural affiliation. We also study the ways in which strategic interventions in public space can afford social interactions between members of groups who would normally live completely separated lives in the same neighborhood. The interventions and art installations by RAAAF are not about prescribing one specific form of use, but rather try to create places that afford a diverse range of spontaneous activities. We feel that an openness to new, marginal or unorthodox types of use are an important prerequisite for interesting public spaces. This is why the majority of RAAAF’s interventions solicit unexpected or unconventional forms of use.

### Philosophical research: Skilled action and the notion of affordances

The strategic interventions of RAAAF are partly based on the philosophical research on skilled intentionality, in which the notions of *skilled action* and *affordances* play an important role.

Understanding skilled action in everyday life is crucial for understanding public domain because daily uses of public space shape the majority of people’s public experiences. Furthermore, these actions contribute to the continuation of sociocultural practices (Van Dijk and Rietveld 2017) and the familiar dealings that groups maintain with particular meaningful places. A characteristic of such familiar dealings with certain places is the fact that a particular environment can evoke a distinct way of going on for individuals belonging to a particular group; a distinct pattern of behavior (Nio et al. 2008, p. 15; see also Rietveld and Kiverstein 2014 on place affordances). An interesting aspect of this phenomenon is that this environmentally evoked activity is oftentimes skilled in an original, situation-specific way (from the perspective of the own group) and does not need to involve any reflection.

Certain embodied skills have a key role in this (Rietveld 2008a, b). An activity like riding the bike is an example of such embodied know-how. While you are riding your bike you will, without reflection, take all kinds of aspects of your context into account: the slipperiness of bridges, (some) traffic rules, an awareness that taxis and trams form a larger threat to you than other traffic does, the possibility of tourists suddenly opening their taxi’s doors without checking if somebody is about to pass them, etc. How is it possible that your action of riding a bike can take all these things into account without reflection? Since the answer to this question is very complex, it is prudent to investigate such instances of skilled action not just from a philosophical point of view, but from a neuroscientific and psychological standpoint as well (for such an integrative approach see Rietveld, Denys and Van Westen, in press; Bruineberg and Rietveld 2014; Rietveld 2008c). This way, insights from these (and other) disciplines can inform and supplement each other and eventually contribute to a thorough understanding of the phenomenon of skilled action in our daily practice.

A philosophical analysis (Rietveld 2008a, b, c; Rietveld, Denys and Van Westen, in press) of skilled daily actions, from the perspective of embodied or ecological-enactive cognition, shows that we can understand these actions in terms of engaging with relevant *affordances,* a term originally coined by the ecological psychologist James J. Gibson (1979, also see Michaels 2003; Chemero 2003; Rietveld and Kiverstein 2014; Van Dijk and Rietveld 2017). Affordances are the possibilities for action provided to us by the environment. A person who has acquired a certain skill has become attuned to relevant affordances (in a particular context) on the basis of their history of past engagements (Rietveld 2008a; Merleau-Ponty 2002/1945). A frequently used object will become a familiar object and so to speak “solicits” (Dreyfus and Kelly 2007; Rietveld 2008a) — i.e. *invites* (Withagen et al. 2012) — interaction. In their respective particular contexts, a chair invites sitting, your bed solicits sleeping, and a glass of water invites drinking from it.^4^ Relevant affordances do not ignore the skilled body, but rather prepare it for action. Without the interference of explicit deliberation, they can solicit and drive a skilled action.

It is important to realize that embodied skills are also at the basis of smooth and adequate social behavior. Pre-reflexive invitations to act are not only afforded by familiar and trusted objects or places, but also by other people. In the latter case, one is dealing with social affordances: possibilities for social interaction or sociability provided by the environment. The sight of a sad friend affords consoling him or her, a colleague at the coffee machine solicits small talk, and an extended hand immediately prepares the body for shaking it.

The role of affordances is important within the context of the real-life experiments undertaken by RAAAF such as *The End of Sitting* (Rietveld 2016; Rietveld et al. 2015). It underlies the possibility of employing specific interventions in public space to solicit spontaneous interactions between people belonging to different socio-cultural groups or subcultures.

## The roles of familiarity and trust for the social fabric of the city: The case of Amsterdam

Amsterdam is historically known as a city of tolerance and diversity. From early on, this old and influential port city attracted people from all over the world. Philosophers like Spinoza, whose books were forbidden across Europe for over two centuries, felt at home in Amsterdam because of its open climate. Until 20 years ago Amsterdam was a leading example of sexual freedom, gay marriage and progressive drugs policy. Currently, Amsterdam is still one of the most culturally diverse cities in the world. At the same time, the City of Amsterdam is now ‘building’ spatial segregation by realizing 50,000 new dwellings: the largest city expansion in decades will be realized on the North bank of the River IJ. This postindustrial area with abandoned wharfs is currently home to an ageing population of old working-class families combined with a younger population of second and third generation immigrants. During the general elections of 2017 the populist right wing Freedom Party (PVV) became the most popular party in Amsterdam North. The existing — relatively poor and low educated — population will be forced to live together with 80,000 new expats and rich, highly educated people who will inhabit the 50,000 new luxurious houses in North. According to the currently existing plans new public space in this area will become ever more commercialized and consumptive, in line with the trend in Amsterdam’s inner city. This puts enormous pressure on the public domain in Amsterdam North. It represents a process of rapidly increasing segregation which is a deathblow to the social fabric of the city of Amsterdam.

These developments are similar to what can be seen in London, Paris, New York and many other big cities across the globe. All of these cities exhibit increasingly segregated neighborhoods in which people from different socio-cultural groups no longer have any contact with each other. One major question for cities in the twenty-first century is: how do we invite people from different socio-cultural backgrounds to *meet*? How can strangers become *trusted familiar strangers*?

The answer to this question can be found in the quality of public spaces, which lies at the basis of familiarity with certain places, as well as the trust in people from different socio-cultural groups. This familiarity and trust are crucial for the social fabric of the city, and good public spaces are imperative for achieving this. In their study of ethnic and social diversity in the so-called Western Garden Cities in Amsterdam New West, authors Ivan Nio, Arnold Reijndorp and Wouter Veldhuis emphasize that this familiarity with others forms the core of well-functioning public spaces that facilitate, and realize, interactions between individuals, and underline the importance of people becoming “familiar strangers” to their neighbors (Nio et al. 2008, p. 134; cf. Jacobs 1961). It is important to note that a ‘stranger’ here is explicitly not defined from one ‘default’ perspective (like the ‘original inhabitant’ who might regard foreign immigrants from different ethnic backgrounds as ‘strangers’), but rather constitutes a relation: in this case, a ‘stranger’ entails somebody from a different socio-cultural or subcultural group to one’s own; somebody adhering to different patterns of behavior than one’s own. Based on their study of the role of public facilities in the Western Garden Cities, Nio et al. suggest that an absence of such *public familiarity* (Blokland-Potters 2005) often results in a fear of the unknown that renders the sharing of a space with strangers a threatening experience (Nio et al. 2008, p. 84).

One of the scientific questions arising here is whether it’s meaningful to regard this smooth way of dealing with strangers as a special kind of embodied skill that people could develop, in which some could even become ‘experts’. Generally speaking, acquiring a certain type of know-how is an implicit affair. Might it be possible that in order to acquire a social skill like dealing with strangers from a different socio-cultural group, a minimal but frequent form of physical contact is necessary? Interestingly, Voestermans and Verheggen (2013, pp. 201–208, 310–311) introduce the term of *bi-cultural competence*, to characterize people who are able to switch easily from dealing with one subculture to dealing with another. Crucially, the notion of bi-cultural competence suggests that it is simply a kind of skill that one can acquire given the right circumstances.

An empirical question connected to the hypothesis of minimal required contact is whether a very minimal form of engagement, like observing somebody from outside a café or in the queue at the baker’s, is sufficient for developing the social skills necessary for engaging appropriately with strangers from a different socio-cultural background. This assumption seems to contradict influential socio-psychological literature (Allport 1979/1954), which suggests that social cohesion is only possible when individuals from different groups make a shared effort in achieving a common goal and, moreover, for which these groups have to be dependent on each other for reaching this goal. The idea behind this is that mutual preconceptions diminish when the members of the different groups get exposed to each other under these (and some additional) conditions (Allport 1979/1954): they get to know each other this way.

### A lack of experienced social cohesion understood as a lack of familiar strangers

Against this background, it’s interesting that an influential report on the social fabric in Dutch neighborhoods and cities, just like Nio et al. (2008) and Voestermans and Verheggen (2013), suggests that a more basic and minimal form of interaction plays a role that is at least as important:

“The call for social control through increased social cohesion concerns much more the lack of trust with respect to strangers in the environment, than it does the wish to get to know one’s neighbors […]. A lack of social cohesion is *a lack of public familiarity*. People are unable to socially account for others — for strangers. This results in them no longer feeling safe and not having a grip on their street, neighborhood or city, of which they feel it no longer belongs to them.” (Dutch counsel for Traffic, Spatial Planning and the Environment (VROM-Raad/VROM-Council), 2006, p. 69, translation and emphasis ours)

An urban neighborhood with a good social fabric therefore does not necessarily mean that everybody has to know each other personally in order to experience a feeling of security or ‘being at home’ (Blokland-Potters 2006). When people repeatedly are exposed to and observe each other (for example on the street, in a bar, at the store or by a bus stop), there’s a very minimal form of physical contact which affords, without them knowing or speaking to each other, a particular form of (public) familiarity to build up (Nio et al. 2008). A lack of social cohesion is, according to the VROM-Council (2006, p. 58), primarily to be understood as a lack of experienced public familiarity; or, more precisely, as a lack of *familiar strangers* in the street or neighborhood (Jacobs 1961; Reijndorp 2004). Neighborhoods with a high turnover in (or, like in the case of Amsterdam’s new neighborhood: a sudden massive influx of) inhabitants are particularly vulnerable to this problem.

Moreover, due to the increasing heterogeneity of the population, everyday securities have disappeared. A breakdown of regularities in the ways of doing things or “ways of going on” that were previously taken for granted, can undermine the certainties on the basis of which people make sense of the world (Rietveld 2008a, c; Wittgenstein 1953, 1975). Such certainties are the basis of basic trust in things and people (Rietveld 2008c, De Haan, Rietveld and Denys 2013). The everyday securities used to be connected to the self-evident or taken-for-granted character of the socio-cultural practices and relevant affordances that scaffold skilled action. The subsequent disappearance of these certainties has led to insecurities about what is — and what isn’t — appropriate behavior:

“[We used to know] many by face, and roughly had an idea about who was living where. They were, in the words of Jane Jacobs, familiar strangers to each other. Due to the rapid change of the population this familiarity is diminishing. Not only do people no longer know as well who lives where, but there is moreover *confusion regarding ways of dealing with each other, which used to speak for themselves*. This isn’t so much the result of a lack of involvement or increase in anonymity and individualization, but rather the effect of the departure of familiar neighbors and their replacement by new unknown persons.” (VROM-Council, 2006, p. 58, translation and emphasis ours)

Interestingly, the VROM-Council seems to be optimistic about the prospects of improving the social fabric of neighborhoods and cities. Among other things, it requires places and facilities where people from different socio-cultural groups can meet each other, or at least can simultaneously dwell. The latter prerequisite is based on the earlier explained idea that people who frequently see each other can become *trusted familiar strangers* to each other. Above we saw that we come to trust people who are a part of our familiar everyday environment because they are sources of regularities and meaning in the world. How do you unite people who tend to become more and more segregated and estranged from each other? Or better, how do you increase the chances that they become “trusted familiar strangers”?

## “Trusted strangers”, a real-life thinking model for 2025

In 2025 Amsterdam will exist for 750 years. The city’s ambition is to celebrate its liberal heritage. Taking into account the urgent need for good public domain in and around Amsterdam North, along with our own research of and views on how to create such spaces, RAAAF has responded to this ambition by introducing a new project in collaboration with Atelier de Lyon: in the water city of Amsterdam, they give physical shape to the endeavor of turning people into ‘trusted familiar strangers’ to each other in the form of a temporary floating park called *Trusted Strangers | New Amsterdam Park (N.A.P.)*. The animation movie Trusted Strangers is an integral part of this article and can be viewed online through the following URL:

https://vimeo.com/205663543 (password = welcomestranger); please turn on your computer’s sound for the best experience.

The opportunities provided to the city by water have always shaped the form and culture of Amsterdam. *Trusted Strangers | New Amsterdam Park* will become a new public domain that involves and unites diverse social cultural groups from all over the city, and places them right next to each other. In New Amsterdam Park (N.A.P.), people will come with their own identity and feel invited to express it. Highly unexpected encounters occur, fostering interactions between people who would usually never meet: the hip youngsters will come across groups of old first-generation immigrants that have been living in Amsterdam for two times their age, skaters will speed past birdwatchers who have been camped out at their spot for hours, children playing in the petting zoo meet goths from the dark barge across the water, and yuppie joggers will literally run into squatters who are hosting a vegan picnic. N.A.P. becomes a ‘real life thinking model’ for the future of public domain in Amsterdam and other global cities all over the world.

### Social affordances & subcultures in New Amsterdam Park

On the river IJ in front of the NDSM wharf, where enormous ships used to be built, a large fleet of barges will be docked: the basis for a new floating park. A grid of 24 large barges (each 80 m long, 11 m wide and 6 m high), will offer the necessary shelter for a hidden water world on the raw river IJ. Twelve of these barges will be temporarily occupied by twelve different socio-cultural groups. The members of each one of these socio-cultural groups have shared interests and manifest shared patterns of behavior, which can be broadly defined as ‘subcultures’.

An important starting point for the realization of a public domain that could cement social cohesion between these groups is MET’s insight that “the social universe is not human-centered but activity-centered, and activity is a hybrid state of affairs.” (Malafouris 2013, 149). Above we saw that in MET and SIF activity cannot be understood independently of the affordances offered by the material environment. This means that the park won’t just bring people from different backgrounds together in one generic ‘passive’ space. Rather, it will invite visitors to explore places where people from different backgrounds engage in activities with both each other and the material environment.

This is also the idea behind the other twelve barges of the park, which aren’t occupied by a dedicated subculture. Instead, they will provide landscapes of *social affordances*; possibilities for social interaction provided by the environment. The Campfire Barge, for example, invites the gathering of people of different subcultures who like to be warm (and who wouldn’t?), while the Panna Barge attracts people from different sociocultural groups who like to play soccer. Crucially, RAAAF will make sure that the barges with social affordances are made to be *attractive for people from several socio-cultural practices* and will generate new patterns of behavior and invite surprising spontaneous interactions. Around the campfire, a prototypical social affordance in the history of mankind, a few goths may find themselves hanging out with some Surinamese people who originally came for the Kwaku barge. With its 24 barges the park becomes a condensed city floating on the water. The possibility to also observe groups of ‘strangers’ and subcultures from a distance is essential to become familiar with their ways of doing things in order to become *trusted familiar strangers*.

### Labyrinthian structure of N.A.P. invites spontaneous meetings

Generally speaking, N.A.P. will consist of three different types of spaces: the diverse worlds inside the barges, the water streets and water squares in between the barges, and the overhead routes. Contrary to the high quays of the Amsterdam canals, the barges let people get in close contact with the water, allowing them to lean their feet into the water while contemplating. The low quays make it easy for visitors to fish, swim or dock a boat. The omnipresence of the water’s reflection deepens the parks spatial experience.

One of the key aspects of the New Amsterdam Park is that all spaces are to be freely accessible to the public. The open character of the park ensures that visitors can roam freely. This possibility of roaming freely is important because it allows people to over time explore more and more aspects of the New Amsterdam Park. Recent work in theoretical computational neuroscience shows that people have a tendency to gradually explore larger and larger aspects of their ecological niche (Kiverstein, Miller and Rietveld 2017). The aforementioned concept of metaplasticity offers a dynamic perspective of how people will over time appropriate their environment: the architecture of the park is designed in such a way that we expect people, who at first naturally gravitate towards the barge that aligns with their own interests and subculture, will over time be responding to more and more of the social affordances offered in this rich landscape due to the allure of the social affordances and the spatial character of the park, which is designed by RAAAF | Atelier de Lyon to invite the exploration and visiting of other barges. The park is thus not only designed to offer a wide range of very different affordances for many different sociocultural groups, but it also makes sure that it is appealing and inviting to people to explore other subcultures due to the park’s ambitious and unconventional design. Over time the members of the different subcultures will feel attracted to roam further and further through the park. In other words, the perimeter of people in the park will increase.

When entering the park by the pedestrian routes overhead, visitors look over the old shipyard and the typical Dutch clouds blowing over the river IJ. From this high vantage point, people will get a first impression of the diversity of the park and its social affordances and subcultures. Smoke is rising up, palm trees emerge, 3D street art constructions pop up, a grass hill rises over the edge of one of the barges, and many other structures and people are popping out of the massive iron barges floating on the water. The routes on top are an easy and safe way to observe and explore the subcultures down below from an appropriate distance. Instead of straight utilitarian pathways, hundreds of small winding routes and shortcuts through the park generate a vibrant sequence of spatial experiences. Moreover, they ensure that people cannot just go straight to their intended destination, but are always exposed to a few other barges containing different sociocultural groups while crossing the park. Sailing through the water streets by boat, or walking through the dense labyrinth of barges, staircases, bridges and routes overhead, generates an optimal number of informal meetings, confrontations, exchanges and gatherings.

Walking up and down through this labyrinth results in a vibrant sequence of spatial experiences. Seemingly impossible meetings and observations will occur. While amateur botanists are observing the wide variety of plants, they are themselves observed by people in boats passing by and watching them through peepholes in the water streets. Observing and being observed by “the other” is made possible by the material environment of the park (port holes, cut-troughs and the meandering overhead pathways all contribute to this), and is essential to the culture of this park. These possibilities for observation increase the likelihood that strangers become *trusted familiar strangers*. Kids in the Japanese Petting Zoo will meet goths who are programming and gathering in the barge next to them. People drinking in the Moroccan Mint tea garden suddenly become voyeurs while observing the practices of their next-door neighbors in the “Harry on Heels Leather Barge” through small portholes.

While sailing to the cliché ‘Tulips of Amsterdam’, one has to disembark at the docking station in front of Robodock barge, resulting in an unexpected meeting with the obscure world of robotic art and fire, deliberately affording people the chance to leave their comfort zone and experience something completely alien to them: in the Robodock barge people with various backgrounds gather to create a gripping world of arts, robotics and fire. The New Amsterdam Park allows its visitors to experience the results of these kind of new and unconventional collaborations in their city. The configuration of the barge grid, combined with routes through water and over land, ensure that people cannot simply stick to their familiar surroundings. Thanks to this structure they will dynamically encounter many different worlds and subcultures.

### Microclimates and trusted familiar strangers

In North European port cities like Amsterdam, it is essential to introduce a human scale within the inhuman scale of the colossal postindustrial port areas. The fleet of 24 barges with its six-meter-high walls provide shelter from the wind on the open water. The spatial structure creates microclimates that are highly suitable for a wide range of atmospheres, meetings and manifestations, such as Riverside Gallery and Classical music barge, or the growth of the unusual vegetation found at the Jungle Magistra barge. In between the barges, water streets and aquatic alleys, one finds water squares that generate activities such as the Palm Beach swimming pool. These open spaces also invite people to project their own thoughts and ideas on the park.

Some spaces such as the Sand Barge, Palm Beach, Camp Fire, Japanese Petting Zoo, and the sloping Grass Hill are included in N.A.P. to also provide social affordances that are very easily accessible, designed to lure in people and meet each other. These spaces appeal to all kinds of sociocultural groups and people of all ages, and can be used to play or relax in. Grass Hill acts as a canvas where the city gathers in all of its diversity: it offers a sloping soft field to lie down in with others while watching the morning sun rise over the historical city center, or to watch it set above the gigantic cranes in the port of Amsterdam. Its attractive location and inviting design solicit all kinds of people to leave their ‘own’ subcultural barge and join others in a common activity. The Sand Barge has a similar potential: sand is a compelling social affordance where children and parents from different subcultures can meet. These barges are designed to appeal to a wide audience and provide the best views. Their inviting character helps limiting the phenomenon of people sticking only to their ingroups, thus bringing people of different background together in the same barges.

By sharing unique experiences with strangers, chances for spontaneous interactions increase and, moreover, one can come to see the similarities with the ways in which other people go on. This increases the chances that others turn into trusted familiar strangers. The large field of grass becomes the canvas on which all groups in the city can meet. Thanks to the many social affordances it offers, N.A.P. will provide hundreds of similar situations where even people from the most outspoken subcultures can easily become trusted familiar strangers in a relaxed way. Social affordances will change with the seasons: while summer affords the youth to jump from the barges into the IJ, winter allows for an Ice-skating Barge and a Hot Spring Barge.

### Responsibility and engagement through social affordances

The park is not just an invitation for the free consumption of space, but entrusts a shared responsibility to people from numerous socio-cultural groups / subcultures. This brings a new kind of engagement to the public domain. The Campfire Barge in the park is an important meeting place in cold seasons and during the evenings, resulting in many possibilities for social interaction. This is a good example of how the environment’s social affordances are created by both the material and the social aspects of the park: when it gets colder at night, people will automatically leave their own, more familiar environments to flock to the warm flames of the impressively large fire. Feeling too cold at night is something all people share, and therefore this barge will be attractive to everybody. When gathered round the fire, further possibilities for interaction with people from other subcultures present themselves. For example, people will be solicited to have a chat with the person standing next to them, or tend to the fire by jointly adding wood or poking it.

Some of Amsterdam North’s most notorious figures will be asked to keep the fire burning while visitors share the comfort of the flames. By giving the ‘leaders of the pack’ this responsibility, the park curators ensure that possibly disturbing group behavior will normally be prevented or directly corrected by peers. The group can become proud of being a host for many other groups by offering a warm campfire at night and during cold seasons.

The way in which these possibilities for social interaction are afforded by a particular sociomaterial environment, and can be made attractive by providing the right solicitations (like only offering warmth at the campfire barge(s) and nowhere else), is a nice illustration of how concepts from MET like ‘enactive sign’ and ‘material agency’ (Malafouris 2013) work in the context of SIF. The above examples show how the many social affordances of the park are indeed *enacted* through people’s (material) engagement with the environment: it is only through their engagement with the campfire that people come across the social affordances. Only within the shared engagement with a common enactive sign – the fire that provides warmth – a social connection is established. As previously noted, this is an aspect of the *entire system* ‘brain-body-landscape of affordances, given the fact that “agency and intentionality may not be innate properties of things, but they are not innate properties of humans either; they are emergent properties of material engagement.” (Malafouris 2013, p. 49)

### A diverse program that changes over time

A park calendar will ensure that the park offers a year-round program of activities, ensuring a corresponding continuous generation of social affordances. Various initiatives will be invited to host a large diversity of subcultures, public facilities, as well as a carefully selected park program. Year-round N.A.P.’s curatorial team will actively design the social affordances and invite subcultures to the park. Some social affordances, like the camp fire barge for example, are relevant all year long, others only during certain periods of the year, such as the ice skating barge for example. Temporary ownership of the different barges by different subcultures for a given period (from one month up to a few years) guarantees that the city will have a diverse range of park programs over time. It also prevents a situation where people get all too accustomed to a particular situation, which might cause them to stop exploring the new possibilities of the dynamic environment that the park offers. Unlike most conventional public spaces, N.A.P. will be a constantly changing environment. This dynamic character prevents people from resorting to fixed patterns of behavior. The changes in Amsterdam’s weather throughout the seasons will be an incentive to change programs during the year: a place like the Palm Beach Barge will be transformed into the Hot Spring Barge in wintertime, while the Tulip Barge can be easily replaced by the Ice Skating Barge. Although the barges will be claimed by different subcultures (partly in response to an invitation by the park curators, partly spontaneously), the principle of open access combined with the specific spatial structure affords visiting the different barges as a guest, and will sometimes lead one to spontaneously participate in its activities. The barges can be uncoupled regularly and rearranged within the grid, resulting in new combinations of social affordances and different subcultures ending up close to each other. In N.A.P. the ‘comfort zone’ doesn’t exist.

## Conclusion

Both in Material Engagement Theory and in the Skilled Intentionality Framework, intentionality is understood as an aspect of the entire system ‘brain-body-landscape of affordances’. N.A.P. shows how embodied/enactive cognition and Lambros Malafouris’ work on Material Engagement Theory can be made concrete by thinking in terms of embodied minds situated in a rich landscape of affordances, including social affordances. N.A.P. is an enactive manifesto for a new kind of maritime public domain in Amsterdam. The spatial design of the floating park solicits many short and spontaneous interactions between people who are from different sociocultural groups but share an interest in the same kind of social affordance. Moreover, the floating park is highly flexible, making it extremely suitable as a testing ground for exciting new public domain. We believe that the basic idea of creating social affordances for social cohesion offers a thinking model for new public domain all over the world.

## Footnotes

1 Since affordances are aspects of the ecological niche of a kind of animal, they thus always figure in a “setting of environmental features” (Gibson 1979, p. 129). This environment is characterized by both social and material aspects, and can be best understood as one *sociomaterial* environment offering many different possibilities for action, including possibilities for social interaction: “Looking around one will notice things and people offering multiple possibilities for action. Sitting in the train for example, I can notice the possibility to drink from a bottle of water, talk to a fellow traveler or return to writing this paragraph. All these affordances belong to a wider socio-cultural context (Hodges and Baron 1992; Costall 1995; Ingold 2000; Heft 2001; Rietveld 2008a)” (Van Dijk and Rietveld 2017, p. 2)

2 See Kiverstein & Rietveld (2012) and Rietveld & Kiverstein (2014) on “place affordances” and Heft’s (2001) discussion of Barker’s (1968) behavior settings.

3 Thanks to Simone van Saarloos for pushing us to make this explicit early on in the paper.

4 One of the reviewers was correct to note that some affordances can invite even though they are not frequently encountered, a lion for instance. To understand the impact of dangerous animals like lions and snakes on humans, Malafouris’ (2013) concept of metaplasticity will be useful because we should not just look not at an individual’s history but also the history of interactions of human ancestors with their environments and the practices these activities gave rise to.
